# Individual and Temporal Variation in Habitat Association of an Alien Carnivore at Its Invasion Front

**DOI:** 10.1371/journal.pone.0122492

**Published:** 2015-03-27

**Authors:** Claudia Melis, Ivar Herfindal, Fredrik Dahl, Per-Arne Åhlén

**Affiliations:** 1 Department of Biology, Centre for Biodiversity Dynamics, Norwegian University for Science and Technology, Trondheim, Norway; 2 Department of Ecology, Swedish University of Agricultural Sciences, Grimsö Wildlife Research Station, Riddarhyttan, Sweden; 3 Swedish Association for Hunting and Wildlife Management, Öster Malma, SE-61191 Nyköping, Sweden; Università degli Studi di Napoli Federico II, ITALY

## Abstract

Gathering information on how invasive species utilize the habitat is important, in order to better aim actions to reduce their negative impact. We studied habitat use and selection of 55 GPS-marked raccoon dogs (30 males, 25 females) at their invasion front in Northern Sweden, with particular focus on differences between males and females, between movement states, and between seasons and times of the day. Daily movement pattern was used to classify GPS-locations into dispersing and settled. We focused on both anthropogenic and natural landscape characteristics. Since we did not have any *a priori* knowledge about the spatial scale of raccoon dog habitat selection, we first assessed how landscape characteristics of random points changed with distance from the GPS-location they were paired to. Because changes in habitat use became less pronounced at approximately 5 km for all variables, we focused on habitat use at two spatial scales: fine (500 m) and coarse (5 km). Habitat selection was strongest at the coarse scale, and reflected the results found for habitat use. Raccoon dogs selected agricultural areas and wetlands, lower altitudes, and shallow slopes, and avoided forests, open natural areas, and areas close to water and roads. There were no differences in habitat selection between males and females, or between movement states. This lack of sexual segregation increases the probability of encountering potential mates during dispersal, and therefore the likelihood for reproduction in new areas. The seasonal and diurnal pattern of habitat use may provide guidance for where and when to aim management efforts.

## Introduction

The success of an invasive species is related to the distribution and abundance of habitat suitable for movement and successful establishment [1,2]. Physical features such as water bodies and mountain ranges, and high levels of human activity may act as barriers to dispersal [3], but the effect of such features on movement varies among species [4] and even between demographic groups within a species [5]. This is often caused by differences in habitat association between life history stages [6]. However, the availability of resources or habitat types also influences the extent to which individuals utilise the resource [7,8], which can vary for instance between seasons [7,9]. Accordingly, detailed knowledge about how an invasive species uses the landscape is required in order to plan management actions.

The raccoon dog *Nyctereutes procyonoides* is a medium-size canid native to East-Asia that was introduced in the former Soviet Union during the first half of the 20^th^ century [10]. Since then it has spread and established viable populations throughout Eastern Europe, the Baltic countries, Poland, Germany and Finland [11] and it is now one of the most successful alien carnivores in Europe [10]. Raccoon dogs are believed to have negative impact on populations of amphibians, ground nesting birds, and aquatic birds, especially in insular habitats [12]. Nevertheless, their role as active predators is debated and firm scientific evidence of their negative effects on bird fauna, *e*.*g*. based on studies conducted before and after raccoon dog arrival or removal, is currently lacking [10]. In Fennoscandia, the raccoon dog is a major concern not only for nature management, but also because it can be infected by several viruses (*e*.*g*. rabies) and parasites (*e*.*g*. sarcoptic mange, piroplasmosis, several type of helminthes infestations), which can all be transmitted to wildlife, domestic animals, and humans [13–15]. Although the raccoon dog still invades new areas in Europe, little is known about dispersal routes and spatial behaviour at the invasion front [10]. To prevent further expansion of raccoon dogs, management actions to detect and eradicate individuals have been initiated in several countries (*e*.*g*. [16]). However, a precise targeting of the actions depends on knowledge of how raccoon dogs at different stages use habitats, and how this varies temporarily at the invasion front. Consequently, there is currently an urgent need for a better understanding of the spatial association of raccoon dogs (*e*.*g*. use and selection of habitats throughout the year in different types of landscapes) at their invasion front.

Raccoon dogs have a very wide trophic niche breadth [17] and their diet varies seasonally with changing availability of food sources [17–19]. They have low sexual dimorphism [20], they are monogamous, and both male and female participate in rearing the pups [21]. The home ranges of the paired male and female overlap almost completely [22]. Accordingly, there are few physical characteristics that should indicate sex-specific habitat associations, and indeed studies from resident raccoon dogs in established populations show similar habitat requirements and dispersal patterns among males and females [23–25]. However, the extent to which this is representative for the invasion front is not clear.

Based on this, we investigated the habitat association of 55 GPS-marked raccoon dogs at their invasion front in Northern Sweden with the aim to: (a) evaluate the spatial scaling of habitat use, i.e. how use of habitat changed based on the spatial scale that was considered, (b) assess whether habitat use and selection varied seasonally, diurnally, between sexes, and among settled and dispersing individuals, and (c) identify important habitats for dispersal and settling where management efforts should be focused. Variation in habitat association was described both by habitat use (i.e. the characteristics of the landscape at raccoon dog GPS-location) and by habitat selection (i.e. how individuals use their habitat relative to the availability of habitat types or other landscape characteristics [26–28]). We expected a higher use of habitats associated with food (*e*.*g*. wetlands, agricultural lands) when settled compared to dispersing, and also a more pronounced habitat selection at the settled state due to the fact that settled individuals are more familiar with what is available. Based on the low level of sexual dimorphism in raccoon dogs (see above), we did not expect to find sex-differences in use and selection of habitats. However, we expected habitats providing cover (i.e. forested areas) and not associated with humans to be used during daylight hours whereas during the dark hours raccoon dogs were expected to be found more often in open areas close to humans, where food resources for this generalist omnivore are more abundant. Finally, we expected seasonal patterns in habitat association to reflect seasonal differences in food availability. Accordingly, we expected wetlands to be more used in spring and early summer (breeding season for birds), and forests to be more used in autumn (high abundance of berries).

## Materials and Methods

### Study area

**Fig 1:**
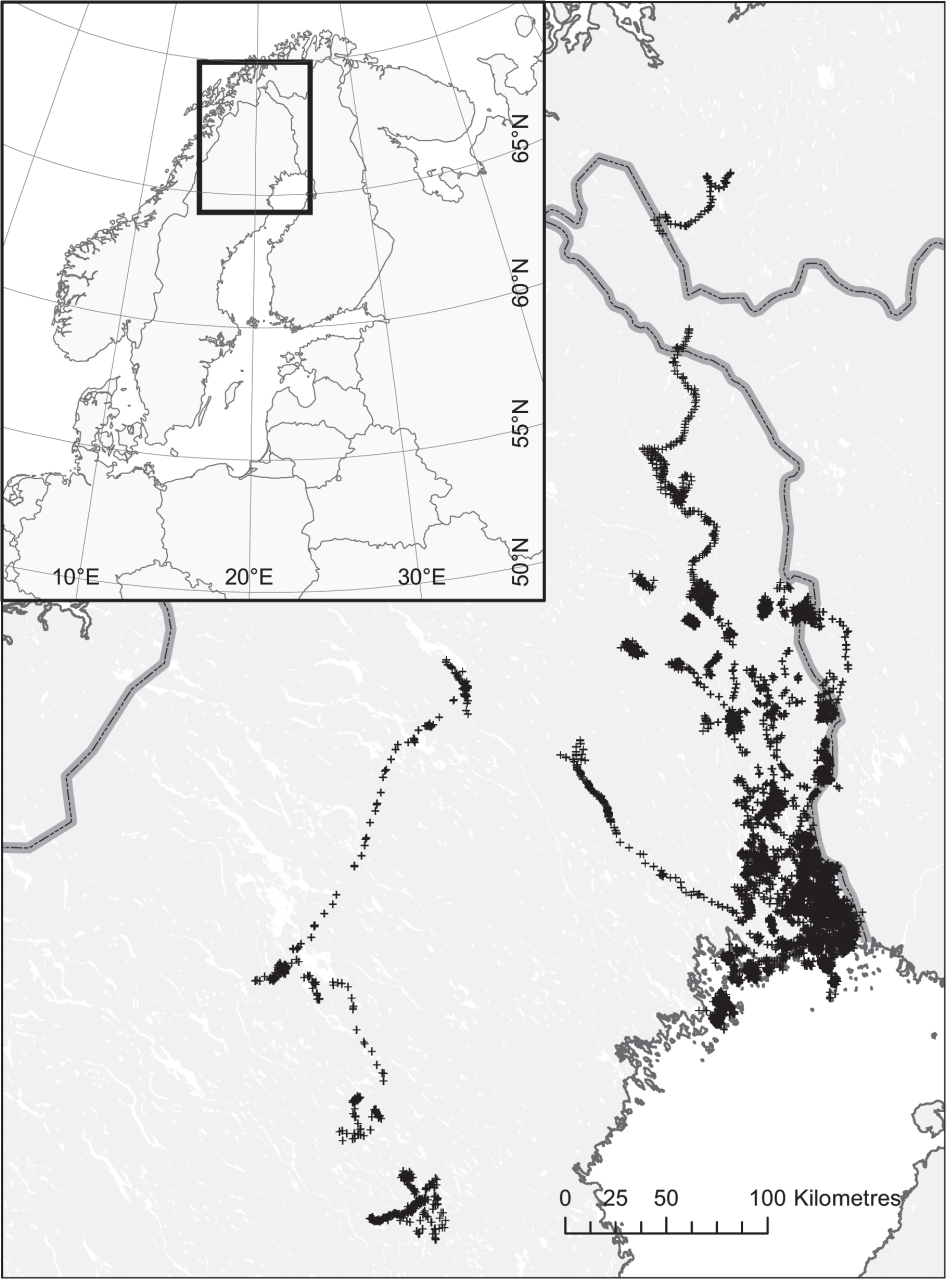
Daily location for 55 GPS-marked raccoon dogs in Northern Sweden. The long-distance trajectories north and west are dispersal events from separate individuals.

The study area is situated within the middle boreal forest region of northern Sweden (Fig 1) and is dominated by coniferous forest intensively managed for timber production, where Scots pine *Pinus sylvestris* and Norway spruce *Picea abies* together with birches *Betula pendula* and *B*. *pubescens* are the dominant tree species [29]. The climate is continental and very variable over the area, with cold to moderately warm summers (mean temperature in July ranging 0° to 15°C) and cold winters (mean temperature in January ranging -9° to -17°C). The annual precipitation ranges from 300 to 2000 mm in different parts of the area. The ground is usually covered with snow from November to April. The topography is characterized by an undulating hilly landscape at the coast, slowly rising to altitudes up to 1500 towards the inland.

### Raccoon dog data

The project was approved by the Swedish Ethics Committee on Animal Research (Dnr A69-08, A111-08, A26-12 and A22-13). For research purposes, we had permission from the Swedish Environmental Protection Agency (SEPA) to use hunting dogs and traps to capture raccoon dogs all year round (Dnr 417-6331-08, Dnr 412-6478-08). Raccoon dog observations, including the ones reported from the public, were followed up by means of infrared/motion triggered cameras (Scout Guard Australia) directed at scent lures and by snow tracking (in winter). When a reliable observation was confirmed, raccoon dog individuals were captured using traps or dogs. All captured individuals were sterilized by vasectomy (males) or oviduct ligation (females) to prevent reproduction but at the same time preserving their normal reproductive behaviour (*e*.*g*. [30,31]). Finally, all individuals were fitted with ear tags and GPS/SMS transmitters (Followit AB).

The GPS/SMS transmitters were programmed to record a location every third hour. There were however some longer gaps in the data, mainly due to failure in obtaining GPS-locations, but also because individuals were recaptured to change batteries and, if necessary, to provide care (*e*.*g*. healing of wounds, treatment against parasites and diseases). Since these data were collected in the framework of a management project, care was provided to keep GPS-collared individuals in good shape so that they could effectively reveal the presence of other raccoon dogs. For this purpose, the animals could also be released into new areas [16]. A recapture event, especially if the raccoon dog was moved into a new area, might affect its behaviour. Moreover, such breaks in the trajectory (i.e. the path described by the animal when it moves) do not represent "natural" breaks. We therefore treated each trajectory between two recapture events as an independent raccoon dog trajectory. Each trajectory was screened for errors following the method described by [32].

We used clustering analyses to assign movement states to the steps (i.e. the straight line between consecutive locations) [33]. The chosen clustering approach uses behavioural characteristics of the steps to separate them into groups that differ statistically [33]. Steps were characterised by step length (the distance between two consecutive locations), relative turning angle (the shift in direction between two steps), and net displacement rate (the increase or decrease in distance from the first location of the trajectory), all based on daily mean locations. The clustering algorithm separated steps into three groups. One was characterised by long and directional movement, henceforth referred to as “dispersing state”. A second group had intermediate daily step lengths but high turning angles, and was designed as “settled state”. A third group with few locations was recognised by very short steps (i.e. within the precision of the GPS-collar, approximately 5–10 m) and high turning angles, and was designed as non-movement state (*e*.*g*. winter sleeping), although individuals might not actually be sleeping but just reducing their activity. Settled and non-movement states were then pooled into one movement state as the number of non-movement steps was low and because individuals in the non-movement state could be considered to be settled (i.e. not dispersing). To include the temporal aspect of movement, all steps within a seven-day period were assigned to the dominating state (i.e. the state represented by 4 or more daily steps). If no state was dominating, the steps were assigned to the settled state. The three-hour interval steps were assigned the movement state of the daily step they belonged to.

Our marked raccoon dogs could not reproduce [16]. We therefore defined seasons based on climate rather than raccoon dog rut, birth, and pup-rearing (*e*.*g*. [34]), and used the following classification: summer (June-August), autumn (September-November), winter (December-February) and spring (March-May). We assigned the solar elevation to the three-hour data set by using the R [35] package maptools [36]. Locations were then assigned a light regime using the following definition: solar elevation < -6 degrees: Night, solar elevation > -6 and < 0: Dusk (before midnight) or Dawn (after midnight), and solar elevation > 0: Day. Due to the high latitude of the study area, we had few locations at night during summer.

### Environmental data

We used the following landscape characteristics: distance to roads, distance to water, elevation, slope, and length of the growing season. The road and water data, and the digital elevation model were provided by the Swedish mapping authority. Roads included all public roads and larger private roads, whereas water data included both larger water bodies such as lakes and large rivers, and smaller creeks. The digital elevation model had a spatial resolution of 50X50 m, and was also used to calculate slope. The length of the growing season was given by the number of days with temperatures above the vegetation growth limit, defined as 5 day degrees, and was delivered by the Norwegian Meteorological Institute with a spatial resolution of 100X100 m.

We used the Corine data set [37] with 100X100 m resolution to describe habitat types. The 44 land cover classes in the Corine data, of which many were not present in our study area, were classified into 6 classes: open water, forest, wetland, open natural habitat (heathland, meadows), agriculture areas, and artificial areas (cities, airports, roads, and other developed and non-vegetated areas). Forested areas could be classified into coniferous forest, broadleaved forests, and mixed coniferous and deciduous forests. We ran the analyses both with each forest type separately, and for all types pooled into one class (forests).

### Defining spatial scale for habitat selection analyses

The choice of the spatial scale at which to study habitat use and selection may depend both on the species and the study area (*e*.*g*. [38]), and it can be related to factors such as scale of movement and heterogeneity of the landscape. We did not have any *a priori* knowledge about the spatial scale at which it would be most appropriate to study raccoon dog habitat selection, and we therefore applied a multi-scale approach in the initial analyses. Accordingly, we generated 8 sets of random points by adding one random point to each GPS-location within the following distances to the GPS-location: 100 m, 250 m, 500 m, 1000 m, 2500 m, 5000 m, 10000 m and 20000 m. This provided us data that could be used to assess how estimates of habitat use changed over the spatial scale. The habitat characteristics at the random points were also used as a measure of availability for the habitat selection analyses (see below). A random point was assigned the same individual identity, sex, season, light regime, and movement state as the GPS-location it was paired to, whereas it had its own habitat type and landscape characteristics. For all points, we calculated the shortest distance to roads and water, and whether a point was inside open water or not. For the other landscape characteristics and habitat types we extracted the value at the point location. Distances to road and water were ln-transformed prior to statistical analyses.

### Statistical analyses

In all analyses we had interdependence between the locations belonging to the same trajectory and individual. We therefore modelled habitat use and selection by a mixed model approach with trajectory and individual identity as random variable [38].

#### Habitat use

We first assessed how habitat use (i.e. the habitat type or landscape characteristics of GPS-locations or random points, [28]) changed with the spatial scale at which the random points were generated. Next we used only the true GPS-locations and investigated whether there were differences in habitat use between males and females, between movement states, and between seasons and light regimes. In addition to main effects of sex, season, light regime and movement state, we also assessed whether any temporal effects varied between sex and movement states by including the following interactions: light regime x sex, light regime x movement state, season x sex and season x movement state. We used likelihood ratio tests [39] to assess any effect of covariates on values of landscape characteristics.

#### Habitat selection

Habitat selection is commonly analysed as the use of habitats relative to their availability [26–28]. A challenge with this approach is to define availability, as this will directly affect the estimate of habitat selection. Individual home ranges are often used for this purpose, but defining home range for dispersing individuals is not straightforward. We therefore chose to use the random points as described above as measure of availability. We chose two spatial scales of availability to represent fine-scale and large-scale habitat selection. Analyses were run separately for the chosen spatial scales. We used resource selection functions (RSF, [27]) to estimate how habitat variables influenced the likelihood that a point was a true location, and not a random point. We first ran separate models for each landscape characteristics. A global model included the landscape characteristic (LC), season, light regime and movement state, but not sex as there was little evidence for sex-differences from the analyses of habitat use (see *Results*, *Factors affecting raccoon dog habitat use*). We also included the interactions between LC and season, LC and light regime, and LC and movement state, as well as three-way interactions between LC, light regime, and movement state, and between LC, season, and movement state.

As a final step, we generated models with multiple landscape characteristics, including only those that had explanatory power in the univariate models. We did this to assess whether the effect of a variable was still retained after accounting for the other landscape characteristics.

For the univariate tests, we used likelihood ratio tests to assess whether the probability that a point was a true raccoon dog location was related to the landscape characteristics, and if this depended on any of the covariates. We used AIC corrected for small sample sizes (AICc, [40]) to evaluate the importance of the explanatory variables in the combined models. Candidate models included all combinations of explanatory variables from the global model. If an interaction was included, the two main effects were always retained in the candidate model. All statistical analyses were run in R version 3.0.2 [35], where the mixed models were run within the lme4 package [41].

## Results

Out of 55 individuals, eighteen had continuous data for more than 200 days. The average home range size (95% MCP) of these individuals was 1168 km^2^ (SD = 3285). However, these data include two long-distance dispersers with annual space use of 4735 km^2^ and 13575 km^2^. When including only individuals with more than 95% of the steps in the settled state (i.e. individuals with settled annual home range, N = 10), the mean home range size was 91 km^2^ (SD = 156). Also here, two individuals showed very large movement between two or three core areas, with home ranges of 454 km^2^ and 302 km^2^. Excluding these two individuals resulted in a mean home range size of 19 km^2^ (SD = 16).

### Patterns of habitat use at multiple spatial scales

**Fig 2:**
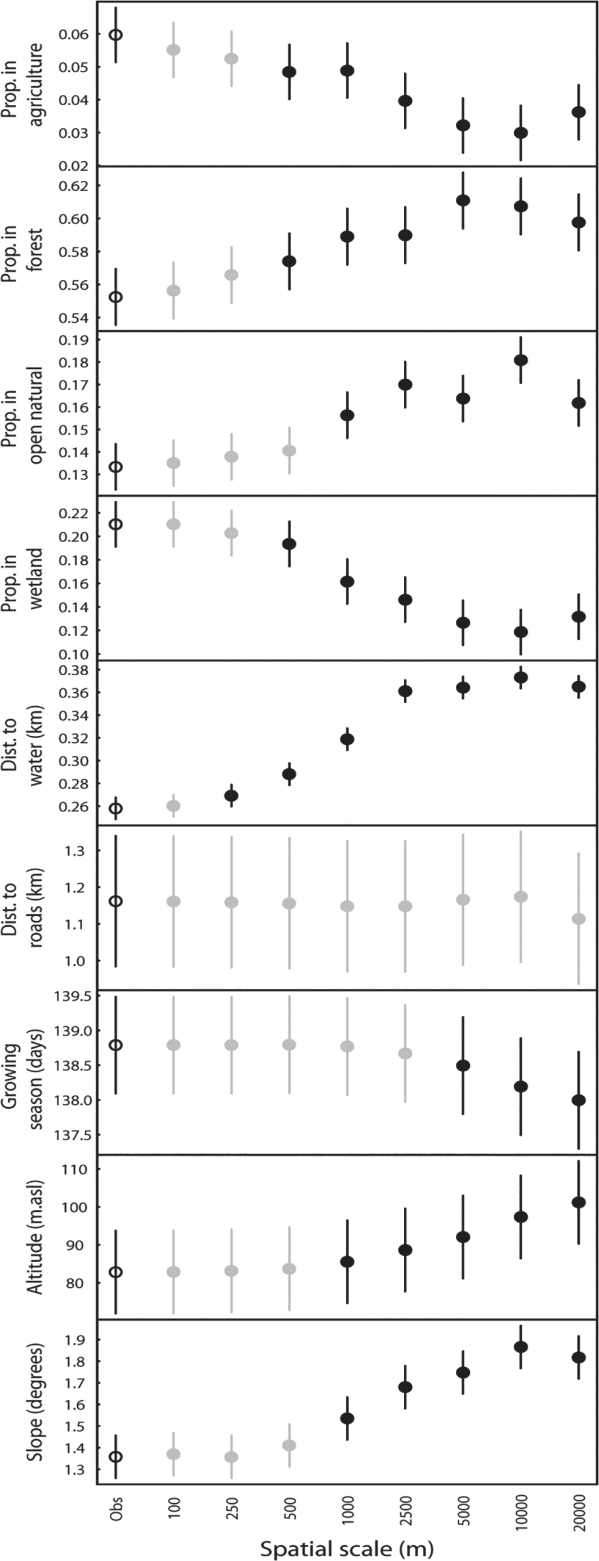
Overall use of habitat types and landscape characteristics by raccoon dogs in Sweden. "Obs" refers to GPS-locations, whereas the spatial scales are points (1 random point per GPS-location) randomly located within a given distance from the GPS-location. Grey symbols indicate that the value at the spatial scale was not significantly different from the values at the GPS-locations whereas black symbols indicate significant differences. Significance was obtained by 95% credible intervals from 10 000 MCMC resamplings of a mixed model with raccoon dog identity and treatment number as random factors. Bars represent standard error of the parameter estimate from the mixed model.

Raccoon dog GPS-locations were most often located in forested habitat (55.2% of all points were in forest, Fig 2); followed by wetland areas (21.0%), open natural (13.3%), agriculture (6.0%), water (4.0%) and artificial areas (0.4%). When we separated the forest into forest types, 44.1% of all points were in coniferous forest, 10.1% in mixed forest, and 2.1% in broadleaved forest. The mean distance to water was 0.26 km (± 0.14 SD), whereas points were on average 1.16 km (± 1.61) from public roads. The length of the growing season was on average 138.8 days (± 6.0) at raccoon dog locations, and the elevation and slope were 82.8 m a.s.l. (± 95.6) and 1.36 degrees (± 1.16), respectively (Fig 2).

When the spatial scale was increased (i.e. the maximum distance from a GPS-location at which random points were generated) the values of the different landscape characteristics changed. For some landscape characteristics the change was quite rapid and there were significant differences between the value of the locations and the random points already at 250 to 500 metres (*e*.*g*. agriculture areas, forest, wetland, distance to water, Fig 2). For other landscape characteristics there was no statistically significant change until the distance was 5000 metres or more (*e*.*g*. distance to roads, length of the growing season, Fig 2).

### Factors affecting raccoon dog habitat use

**Fig 3:**
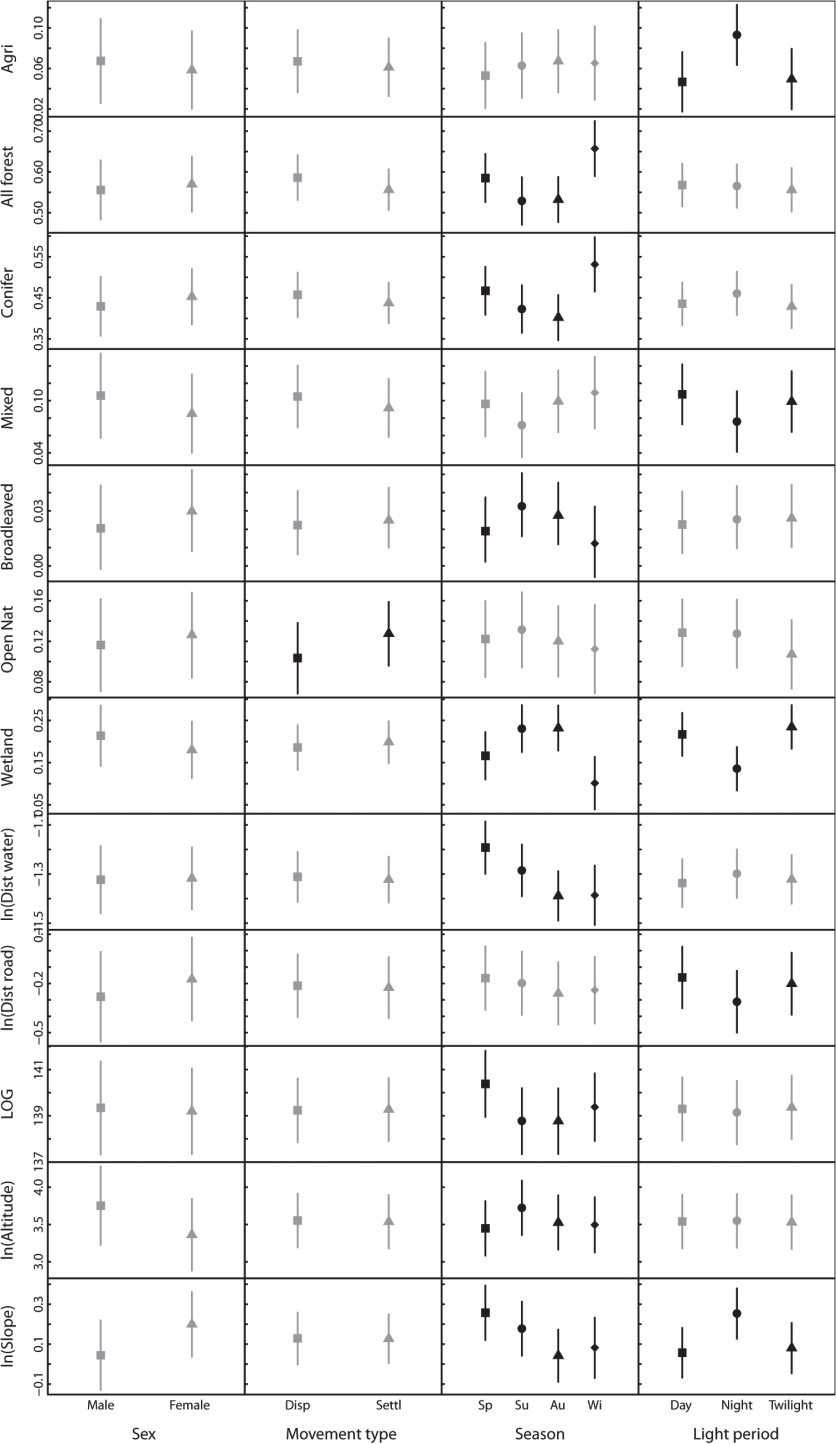
Parameter estimates on habitat use with respect to sex, movement state, season, and light regime in raccoon dogs in northern Sweden. Bars represent 95% credible interval based on 10 000 MCMC resampling of the posterior distribution of the parameter estimates. Grey symbols and bars indicate no significant (*P* > 0.05) difference between the groups, i.e. adding the covariate (sex, movement state, season, or light period) did not improve a null model (including only intercept). Black symbols and bars indicate a significant improvement of the model by adding the covariate.

We found no statistical evidence for differences in habitat use between male and female raccoon dogs (all *P* > 0.195, Fig 3). Consequently, we did not include sex as an explanatory variable in further analyses.

The use of open natural areas was higher for individuals in the settled state compared to dispersing individuals (χ^2^ = 4.95, df = 1, *P* = 0.026, Fig 3). There was also a tendency for raccoon dogs to use forest more during dispersal than when settled (χ^2^ = 3.39, df = 1, *P* = 0.065, Fig 3), but this pattern was less clear when separating between forest types (all *P* > 0.124, Fig 3). For the other landscape variables there was no statistical evidence for differences in use between dispersing and settled individuals (all *P* > 0.331, Fig 3).

There were significant seasonal differences in how habitat and landscape variables were used by raccoon dogs (Fig 3), except distance to road, proportion of agriculture areas, and proportion of open natural areas (all *P* > 0.277, Fig 3). During winter, raccoon dogs used forest more and wetland less than the rest of the year (test of seasonal differences in use of forest: χ^2^ = 20.72, df = 3, *P* < 0.001, wetland: χ^2^ = 33.60, df = 3, *P* < 0.001). We found a similar pattern for coniferous forest as for the pooled forest group (χ^2^ = 21.19, df = 3, *P* < 0.001), and also a significant season effect on the use of broadleaved forests (χ^2^ = 9.88, df = 3, *P* = 0.020), which was opposite to the seasonal pattern of coniferous forest (Fig 3). The use of mixed forest did not differ significantly between seasons (χ^2^ = 7.28, df = 3, *P* = 0.063), but the seasonal pattern was similar to that of coniferous forest (Fig 3). Raccoon dogs were farthest away from water during spring, and closest during autumn (χ^2^ = 18.98, df = 3, *P* < 0.001). The growing season was longer in areas used during spring compared to the rest of the year (χ^2^ = 69.05, df = 3, *P* < 0.001), and during spring raccoon dogs were also at lower altitudes (χ^2^ = 53.38, df = 3, *P* < 0.001) and on steeper slopes (χ^2^ = 18.23, df = 3, *P* < 0.001) compared to the other seasons.

Raccoon dog habitat use varied with light regime, with the greatest difference between night and the two other periods (Fig 3). However, for proportion of forest and open natural areas, distance to water, length of growing season and altitude, there were no statistical significant differences in use between the different light regimes (all *P* > 0.121, Fig 3). Among the forest types, the use of coniferous and broadleaved forests did not vary significantly with light regime (*P* > 0.134), whereas the use of mixed forest was lower during the night compared to day and twilight hours (χ^2^ = 12.82, df = 2, *P* = 0.002, Fig 3). Agricultural areas were used more at night compared to the day and twilight hours (χ^2^ = 36.09, df = 2, *P* < 0.001). Wetland was used least at night compared to day and twilight (χ^2^ = 55.57, df = 2, *P* < 0.001). Raccoon dogs were closer to roads at night (χ^2^ = 25.91, df = 2, *P* < 0.001), and used areas with steeper slopes at night compared to day and twilight hours (χ^2^ = 41.83, df = 2, *P* < 0.001, Fig 3).

For the analyses of interaction effects, we were primarily interested in how sex and movement state affected habitat use, depending on season or light period. However, as there was no evidence for sex-specific habitat use, and the only difference between dispersing and settled individuals occurred in the use of open natural habitats, we did not pursue interaction effects further.

### Habitat selection at two spatial scales

Based on the scale-specific results described in Fig 2, we chose to use random points at the scale of 500 m (fine scale habitat selection) and 5000 m (large scale habitat selection) for analyses of habitat selection.

**Fig 4:**
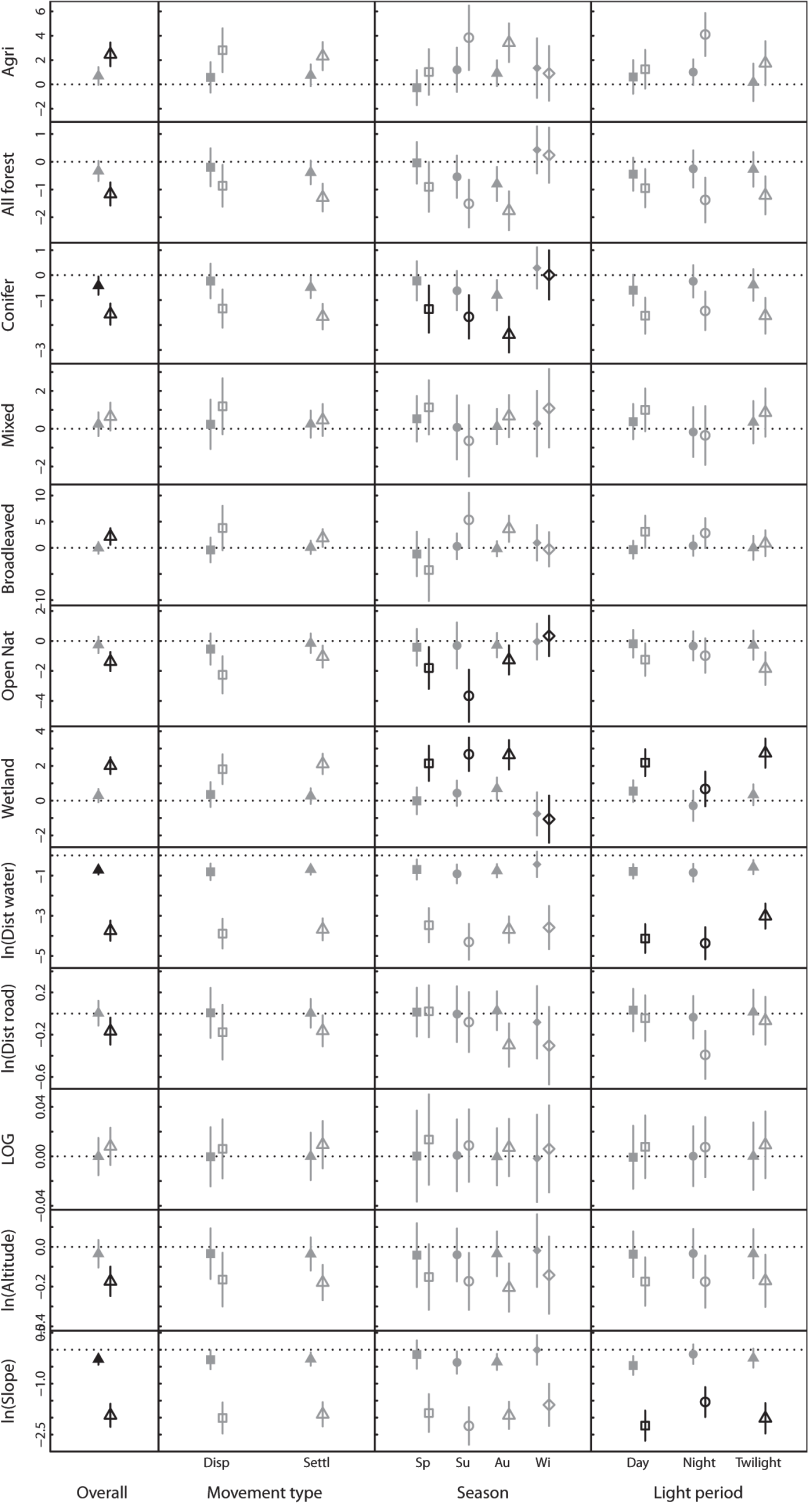
Habitat selection at two spatial scales for raccoon dogs in northern Sweden. Bars represent 95% credible interval based on 10 000 MCMC resampling of the posterior distribution of the parameter estimates. Filled symbols represent habitat selection at the 500 m spatial scale, whereas open symbols represent habitat selection at the 5000 m spatial scale. A positive value indicates that the probability of use is higher for high values of the explanatory variable whereas a negative value indicates that the probability of use decreased with increasing value of the explanatory variable. The first column is the overall habitat selection for all individuals, where black symbols and bars indicate a significant selection (i.e. the 95% CI does not include zero). In the next three columns, interactions between the landscape variable and movement state, season and light period are shown, where black symbols indicate significant difference in habitat selection between the groups at a given spatial scale.

At the 500 m scale, only distance to water (χ^2^ = 45.07, df = 1, *P* < 0.001) and slope (χ^2^ = 12.01, df = 1, *P* = 0.001) had significant relationships to the probability that a point was a true GPS-location, whereas no significant relationship was found for the other landscape characteristics (all *P* > 0.059). The probability that a point was a GPS-location was negatively related to distance to water and to the slope (Fig 4). This suggests that raccoon dogs prefer areas that are close to water and not steep. Adding interactions between landscape characteristics and movement state, season or light period did not significantly improve the models (all *P* > 0.394, Fig 4). This suggests that there is only a weak habitat selection at the 500 m scale, and that it does not differ much between movement states, seasons or light period.

When we added both proportion coniferous forest, distance to water and slope in the same model and ran AICc-based model selection, the highest ranked model included all three variables, with a ΔAICc to the second best model = 1.47. Parameter estimates from the highest ranked model did not differ much from those from the univariate models, suggesting that at the fine scale, coniferous forest, distance to water and slope are important landscape characteristics for raccoon dog.

At the 5000 m scale, the probability that a point was a GPS-location was significantly (all *P* < 0.010) related to all landscape variables except length of the growing season (χ^2^ = 1.09, df = 1, *P* = 0.297) and mixed forest (χ^2^ = 2.99, df = 1, *P* = 0.084), indicating considerable habitat selection by raccoon dogs at the large spatial scale. The probability that a point was a GPS location was higher in agricultural and wetland areas and lower in forests and open natural habitats (Fig 4). This suggests that agricultural and wetland areas were used more than expected, whereas forest and open natural habitats were used less than expected based on their availability at the large spatial scale. Among forest types, coniferous forest was used less than expected, whereas broadleaved forest was used more than expected based on their availability (Fig 4). Moreover, the negative selection coefficients for distance to roads and distance to water (Fig 4) suggest a selection for areas close to such features. Finally, the probability that a point was a true GPS-location was negatively related to elevation and slope (Fig 4), which suggests that raccoon dogs select areas at lower altitudes that are not steep.

Adding the interaction between landscape characteristics and movement state did not increase the fit of the models significantly (all *P* > 0.250, Fig 4).

There were seasonal differences in the habitat selection for open natural (χ^2^ = 13.61, df = 1, *P* = 0.034) and wetland areas (χ^2^ = 25.79, df = 1, *P* < 0.001), and for the coniferous forest type (χ^2^ = 15.37, df = 3, *P* < 0.018), but not for the other landscape characteristics (all *P* > 0.071). Selection against open natural areas was strongest during summer whereas during winter there was no apparent selection or avoidance regarding open natural areas (Fig 4). Wetland areas were selected during spring, summer and autumn, but not during winter (Fig 4). The selection against coniferous forests was stronger in spring, summer and autumn, whereas there was no clear selection for or against coniferous forest in winter (Fig 4).

There was also a short-term temporal variation in habitat selection. Wetland areas were selected more during day and twilight compared to the night (χ^2^ = 11.00, df = 2, *P* = 0.022, Fig 4). Moreover, the selection for areas close to water was strongest during day and night, and weaker during twilight (χ^2^ = 11.45, df = 2, *P* = 0.022, Fig 4). Finally, the selection for gentle slopes was strongest during day and twilight, and weaker at night (χ^2^ = 11.52, df = 2, *P* = 0.021, Fig 4). For the other landscape variables there were no significant differences in selection by raccoon dog among light regimes at the 5000 m scale (all *P* > 0.149).

As a final step we made a combined model with all significant terms from the univariate tests at the 5000 m scale (see Fig 4), but without forest due to the inclusion of coniferous forest and its interaction with season, and broadleaved forest. The AICc-based model selection gave highest support to the full model except broadleaved forest (ΔAICc to the second ranked model = 9.63). The support to the almost full model suggests that the effect of one variable is not caused by its correlation with other variables, and that the spatial distribution of raccoon dog is shaped by its association to multiple landscape characteristics simultaneously.

## Discussion

Based on a unique dataset on movement of an alien species at its invasion front, we have found habitat association supporting predictions based on costs and benefits associated to specific landscape characteristics. As predicted from the lack of sexual dimorphism in physical and behavioural traits [20–25], we did not find evidence for sex-differences in how raccoon dogs used the landscape (Fig 3). There was also only weak evidence for differences between settled and dispersing individuals (Fig 3 and 4); however, we found considerable temporal variation in habitat association (Fig 3 and 4).

The patterns of spatial scaling in habitat use of raccoon dogs (Fig 2) indicated large differences in how different landscape characteristics changed with distance from the raccoon dog location. This can be caused by several mechanisms. Firstly, there may be differences in the spatial autocorrelation of the landscape characteristics. Characteristics that have little variation even over long distances (high spatial autocorrelation) will show similar values even if points are located far from each other. Secondly, it may be related to whether the characteristic affects the distribution of raccoon dogs in the landscape (i.e. whether there is a significant selection for or against the variable). Finally, it can relate to the spatial scale of raccoon dog habitat use. The home ranges of our marked individuals were considerably larger than reported in other areas [22,42,43]. Our study area has rather harsh environmental conditions compared to others, and a larger space use is thus expected [44], which may affect the spatial scale at which habitat selection is observed. This shows the importance of assessing both use and selection at multiple spatial scales when making inference about animals association to landscape characteristics.

The fine-scale habitat selection analyses revealed few significant patterns, suggesting that raccoon dog selection of habitat occurs at a larger spatial scale than 500 m. Indeed, the home range size of settled raccoon dogs can be as small as 100–200 ha [10], and it may be that some of our habitat variables did not have a sufficiently high resolution to detect habitat selection at the very finest scale. It is however worth noticing that the direction of selection coefficients (i.e. whether the coefficient of selection was positive or negative) was similar for the fine and large-scale habitat selection analyses (Fig 4), indicating that raccoon dogs have similar responses to landscape characteristics at the large and fine spatial scale.

The habitat use as measured by landscape characteristics at the GPS-locations showed that raccoon dogs were most often found in forested areas, wetlands, and open natural areas (Fig 2). However, the large-scale habitat selection coefficients for forest and open natural habitat were negative (Fig 4), suggesting that these habitats were used less than expected based on their availability. Moreover, raccoon dogs were overall more frequently found in agriculture and wetland areas, closer to water and roads, at lower altitudes and in gentler slopes compared to what was available at the large spatial scale (Fig 4). This confirms a habitat association of raccoon dogs towards water or wetland areas [42,45], and areas associated with humans (i.e. the agriculture areas [42,43,23]), most likely caused by a higher abundance of food in these areas [10]. The fact that forest was not selected does not mean that it is not important for raccoon dogs. Forest provides cover and contains a range of food sources for raccoon dogs [18]. However, abundant habitats such as forest in our study area are often found not to be selected even if they are used frequently, as a result of the relationship between availability and selection that generates functional responses in habitat selection [40,41]. Accordingly, we cannot exclude forest as an important habitat for raccoon dogs, and the fact that more that 50% of the locations were in forested areas (Fig 2) suggests that a mixture of forest, wetlands and agricultural areas are important for raccoon dogs.

We did not find significant sex-differences in habitat use for any of the landscape characteristics (Fig 3), supporting previous studies on the lack of sex-specific habitat association in raccoon dogs [23]. This was expected, and is most likely related to the lack of sexual dimorphism [20] and the fact that a pair shares the same territory [21]. This lack of sexual segregation increases the probability of encountering potential mates during dispersal, and therefore the likelihood for reproduction in new areas. More unexpected was the lack of differences in habitat use and selection between settled and dispersing individuals (Fig 3 and 4). The only landscape characteristic that showed significant difference between dispersing and settled individuals was open natural habitats (Fig 3). However, this difference was not significant when assessing habitat association by selection for habitat (Fig 4), suggesting that the difference in habitat use was a result of different availability of open natural habitats among dispersing and settled individuals. Accordingly, it appears that landscape association is similar for dispersing and settled individuals, at least for the spatial scales and habitat types addressed in our study.

There was considerable temporal variation in habitat association of raccoon dogs. Some of these patterns were consistent both when looking at habitat use and habitat selection, such as the lower association to wetlands during winter compared to the rest of the year. Other temporal effects were only significant for habitat use, but not for habitat selection (*e*.*g*. the higher use of agricultural areas during night compared to day and twilight, Fig 3 and 4). Still, even if there were some temporal differences between habitat use and selection, the patterns were mainly similar.

When assessing seasonal differences in habitat association by both use (Fig 3) and selection (Fig 4), the overall pattern was a lower association to wetlands and a higher association to coniferous forest during winter compared to the rest of the year. Moreover, raccoon dogs were found further away from water and on steeper slopes during spring and summer. In temperate and boreal areas the raccoon dog is commonly inactive during winter, usually when the air temperature is below—10°C, snow depth is above 35 cm, and day length is shorter than 7 h [46]. Some individuals might also be active during winter, for instance if they have not accumulated enough fat reserves during summer and autumn. In the study area the winter is rather cold which means that water most likely is frozen and that there are limited food resources in wetland areas. In winter, forests can therefore provide food such as berries and carrion [47], shelter against harsh weather and snow cover [48], and better denning sites than wetlands. We also found selection against coniferous forest in spring, summer and autumn, but not in winter. This pattern was opposite to the one found for broadleaved forest, thus suggesting that the difference in food and/or shelter availability between broadleaved and coniferous forest is less pronounced in winter.

The diurnal patterns in habitat association suggested that the largest differences occurred between the dark hours (night) and the two other categories. During the night, raccoon dogs used agricultural areas most and wetland areas least, and they were closer to roads and at steeper slopes compared to day and twilight (Fig 3 and 4). This can be interpreted as anti-predation behaviour, particularly towards humans. In fact humans are one of the most important mortality factors for raccoon dogs in human-dominated landscapes [49,50]. Such behaviour has been reported also for other carnivores [51,52] and has been suggested to be caused by easier locomotion and higher availability of food close to roads, together with lower traffic volume during night. At the same time, prey of raccoon dogs such as frogs and rodents [12] are nocturnal, and the diurnal pattern is thus most likely shaped by both risk avoidance and predation success.

Although we did find evidence for habitat association in raccoon dogs, there was not a very strong and consistent pattern across seasons or during the day, or between dispersing and settled individuals. This has some management consequences. Firstly, it means that actions cannot be targeted only at one or a few specific habitats. The cost to control an established population of raccoon dogs for the nine million hectares of wetlands in Sweden was estimated to be 29.7 million euro per year [16]. Given the close association also to other habitats, the cost of their control could be much higher if they expand their range further. Secondly, the lack of clear habitat association of dispersing individuals suggests that there are no typical movement corridors in which management actions would be particularly efficient at stopping dispersers. Therefore, it seems even more important to prevent further expansion of raccoon dogs, as delaying such actions will most likely result in considerably higher cost.
